# Don't take this lying down: an urgent wakeup call: the weight of diet and lifestyle in the young‐onset colorectal cancer surge

**DOI:** 10.1111/ans.19416

**Published:** 2025-02-01

**Authors:** Maria Kristina Vanguardia, Chen Lew, Thang Chien Nguyen, William Teoh, Vignesh Narasimhan

**Affiliations:** ^1^ Department of General Surgery Alfred Health Melbourne Victoria Australia; ^2^ School of Translational Medicine Monash University Melbourne Victoria Australia; ^3^ Department of Colorectal Surgery Monash Health Dandenong Victoria Australia

The overall decline in colorectal cancer (CRC) incidence in developed nations is overshadowed by a worrying anomaly: a rise in young‐onset colorectal cancer (YO‐CRC), broadly accepted as CRC diagnosed in patients under the age of 50. Currently, about 10–12% of all new diagnoses of CRC are in patients under 50.^1^, ^2^ Projections suggest that by 2030, YO‐CRC would account for a quarter of all rectal cancers.^3^ Additionally, YO‐CRC is projected to become the leading cause of cancer related mortality in those aged 20–49 by 2030.^4^ Locally, Australia and New Zealand have some of highest age standardized incidence rates of CRC in the world.^5^

YO‐CRCs are more often left sided, and present at more advanced stages of disease, with a delayed time to diagnosis. They are also more likely to present with synchronous metastases.^3^ Studies show that about two thirds of YO‐CRCs have seen at least two physicians before being correctly diagnosed, with over 40% waiting at least 6 months after experiencing symptoms before seeking medical attention.^6^ Some studies suggest YO‐CRCs demonstrate more aggressive histopathological subtypes, such as mucinous adenocarcinoma and signet ring pathology.^3^, ^5^, ^7^

Genetics do provide some clues, but its exact impact is unclear. YO‐CRC patients are almost twice as likely to have pathogenic variants (17%–25%), with about half due to mutations in lynch syndrome genes. The significance of the other pathogenic variants and its relation to CRC is unclear.^8^ The role of low‐moderate penetrance variants, and their gene‐environmental interactions, particularly early in life is an area of ongoing research. A positive family history of CRC in at least one first degree relative is reported in up to 30% of YO‐CRCs.^1^

In keeping with the concept of the birth cohort effect, issues such as obesity, sedentary lifestyle and dietary changes have seen major shifts in recent decades. This is in line with a similar increase in YO‐CRC rates across successive birth cohorts. Obesity and the metabolic syndrome are associated with a significantly increased relative risk for CRC. The Nurses Health Study II reported a significantly higher relative risk for CRC based on BMI, with an incrementally higher risk in obese compared to overweight patients. Childhood obesity sadly has seen a 200% rise since the 1960s.^9^ Similarly, sedentary jobs and lifestyle, which have increased over 80% since the 1950s have been shown to render a significantly increased relative risk for YO‐CRC, particularly rectal cancer.^10^ The metabolic syndrome, with its associated conditions (hypertension, hyperlipidaemia, hyperglycaemia) are strongly associated with an increased risk of YO‐CRC, with incrementally increased odds with each additional component of the metabolic syndrome.^11^ Consumption of sugary beverages have similarly been associated with an increased relative risk for YO‐CRC, with an increasing risk based on consumption.^12^

One of the pivotal advances in recent years has been the recognition that the gut microbiome plays a central role in the development of CRC. Bacteria such a fusobacterium species, bacteroides fragiles, pks+ *E. Coli*, salmonella enterica are strongly associated with CRC carcinogenesis through a variety of mechanisms.^13^, ^14^ Overuse of antibiotics in recent years is a growing health concern. Additionally, epidemiological studies support an association between antibiotic exposure and development of CRC. The general theory is that antibiotic usage early in life perturbs the gut microbiome, with possible loss of protective microbiota early in life. This alteration in the microbiome may contribute to pro‐inflammatory and pro‐carcinogenic consequences, which may predispose individuals to YO‐CRC.^5^

The concept of the exposome has also come to the fore in recent years. The notion of the exposome suggests that environmental factors that have seen a similar temporal trend to YO‐CRC over time and potentially alter the gut microbiome contribute to the development of YO‐CRC.^14^ It is likely a complex interplay wherein exposure to exposomal elements such as the westernized diet, red and processed meat in conjunction with other events early in life like exposure to antibiotics, synthetic dyes, microplastics alter the gut microbiome in childhood. The altered microbiome early in life along with ongoing exposure to environmental factors such as obesity through childhood and adolescence causes alterations in gut immunity and inflammation and likely confer an increased risk of developing YO‐CRC to individuals with underlying genetic susceptibility.^9^, ^10^

The causative factors contributing to YO‐CRC are no doubt complex. The growing tide in YO‐CRC has led to several countries, including Australia to lower the screening age to 45. A more holistic examination of environmental influences from early in life is needed to help understand and address risk reduction in YO‐CRC. There remains ongoing research into areas such as understanding gene‐environmental interactions, susceptibility of the gut to carcinogenic exposures at various ages, role of microbiome and inflammation, with ongoing prospective projects such as GUTS, ORIGINS project in progress. The coming years will hopefully offer more insights into better understanding the key drivers and implementation of preventative strategies in dealing with YO‐CRC.
